# Target arterial PO_2_ according to the underlying pathology: a mini-review of the available data in mechanically ventilated patients

**DOI:** 10.1186/s13613-021-00872-y

**Published:** 2021-06-02

**Authors:** Julien Demiselle, Enrico Calzia, Clair Hartmann, David Alexander Christian Messerer, Pierre Asfar, Peter Radermacher, Thomas Datzmann

**Affiliations:** 1 Service de Médecine Intensive - Réanimation, Nouvel Hôpital Civil, Hôpitaux Universitaires de Strasbourg, 1, place de l′Hôpital, F–67091, Strasbourg, Cedex, France; 2 Institut Für Anästhesiologische Pathophysiologie Und Verfahrensentwicklung, Universitätsklinikum, Helmholtzstrasse 8-1, 89081 Ulm, Germany; 3 Klinik Für Anästhesiologie Und Intensivmedizin, Universitätsklinikum, Albert-Einstein-Allee 23, 89081 Ulm, Germany; 4 Service de Médecine Intensive - Réanimation Et Médecine Hyperbare, Centre Hospitalier Universitaire D’Angers, 4 rue Larrey - 49 933, Angers Cedex 9, France

**Keywords:** Hyperox(aem)ia, Traumatic–haemorrhagic shock, Septic shock, Cardiopulmonary resuscitation, Traumatic brain injury, Ischaemic brain injury, Intracerebral bleeding, Acute subarachnoidal haemorrhage, Surgical site infection

## Abstract

There is an ongoing discussion whether *hyperoxia*, i.e. ventilation with high inspiratory O_2_ concentrations (F_I_O_2_), and the consecutive *hyperoxaemia*, i.e. supraphysiological arterial O_2_ tensions (PaO_2_), have a place during the acute management of circulatory shock. This concept is based on experimental evidence that hyperoxaemia may contribute to the compensation of the imbalance between O_2_ supply and requirements. However, despite still being common practice, its use is limited due to possible oxygen toxicity resulting from the increased formation of reactive oxygen species (ROS) limits, especially under conditions of ischaemia/reperfusion. Several studies have reported that there is a U-shaped relation between PaO_2_ and mortality/morbidity in ICU patients. Interestingly, these mostly retrospective studies found that the lowest mortality coincided with PaO_2_ ~ 150 mmHg during the first 24 h of ICU stay, i.e. supraphysiological PaO_2_ levels. Most of the recent large-scale retrospective analyses studied general ICU populations, but there are major differences according to the underlying pathology studied as well as whether medical or surgical patients are concerned. Therefore, as far as possible from the data reported, we focus on the need of mechanical ventilation as well as the distinction between the absence or presence of circulatory shock. There seems to be no ideal target PaO_2_ except for avoiding prolonged exposure (> 24 h) to either hypoxaemia (PaO_2_ < 55–60 mmHg) or supraphysiological (PaO_2_ > 100 mmHg). Moreover, the need for mechanical ventilation, absence or presence of circulatory shock and/or the aetiology of tissue dysoxia, i.e. whether it is mainly due to impaired macro- and/or microcirculatory O_2_ transport and/or disturbed cellular O_2_ utilization, may determine whether any degree of hyperoxaemia causes deleterious side effects.

## Background

Oxygen (O_2_) is not only the final electron acceptor within the respiratory chain, but also one of the strongest oxidizing molecules [1, 2]. Approximately 1–3% of mitochondrial O_2_ consumption is directed towards the production of "reactive oxygen species" (ROS), i.e. the more ATP produced, the more ROS are generated [3, 4]. ROS formation is directly related to the O_2_ concentration [5], so that *hyperoxia* (i.e. breathing inspiratory O_2_ concentrations F_I_O_2_ > 0.21) and the consecutive *hyperoxaemia* (i.e. arterial PO_2_ > 100 mmHg) result in a dose-dependent increase of ROS formation [6, 7]. It is noteworthy in this context that the definition of hyperox(aem)ia may vary as well: while some authors use a threshold PaO_2_ value of 150 mmHg [8, 9], others refer to hyperox(aem)ia as PaO_2_ ≥ 300 mmHg [10–14]. The aggravated ROS formation resulting from increased O_2_ concentrations is particularly pronounced during ischaemia/reperfusion (I/R) and/or hypoxia/re-oxygenation [5]. Nevertheless, the dichotomy for O_2_ holds also true for ROS formation, in that despite their toxic potential ROS are vital players in host defence systems and as signalling molecules [15].

In line with the potential toxicity of increased ROS formation resulting from supplemental O_2_ administration, a meta-analysis of more than 16,000 patients previously concluded that patients with "*liberal*" oxygenation (defined as transcutaneous, pulse oximetry haemoglobin–O_2_ saturation [SpO_2_] median/range 96/94–99%) "*had a dose-dependent increased risk of … mortality*", yet found "*no significant difference in disability, hospital-acquired pneumonia, or length of hospital stay*" [16]. While providing robust data on a large general ICU population, only 8 out 25 studies analysed in this systematic review, however, had included patients undergoing mechanical ventilation. Moreover, the putative impact of the presence or absence of circulatory shock was not discussed at all. Finally, due to the scarcity of the literature available, a conclusive evaluation of some conditions normally necessitating ICU treatment could not be provided either, e.g. trauma-and-haemorrhage and/or traumatic brain injury [16]. Nevertheless, based on this meta-analysis, an expert panel concluded on a "strong recommendation" that when supplemental O_2_ therapy is used, SpO_2_ > 96% should be avoided in all in-hospital as well as prehospital medical patients. Explicitly, uncomplicated, elective surgical patients were not included because the expert panel had not reviewed the issue of peri-operative hyperoxia and surgical site infection, respectively [17].

On the other hand, more recently, in a population of mechanically ventilated ICU patients, a "*conservative*" oxygenation target (defined as SpO_2_ < 97%) yielded no benefit when compared to a "*usual*" oxygenation group [18], and even suggested that "*usual (liberal) oxygen therapy might be preferred*" in the subgroup of patients with sepsis [19]. Therefore, given the U-shaped relation between mortality/morbidity and PaO_2_ [20], this mini-review will discuss the questions *i)* whether there is an "*ideal*" PaO_2_ for ICU patients, and in particular; *ii)* if present, whether it possibly differs according to the underlying pathology. We will only discuss the available clinical data, since the vast majority of experimental models lack standard ICU care, which necessarily limits their translational value. Moreover, as far as possible from the data reported, we will focus on data from mechanically ventilated *vs.* spontaneously breathing patients. Finally, we will try to evaluate the possible impact of the presence or absence of circulatory shock, a condition where *"administration of oxygen should be started immediately to increase oxygen delivery*" [21]. This review will discuss pertinent clinical studies on general ICU populations and in the emergency department (ED), acute respiratory distress syndrome (ARDS), sepsis and septic shock, trauma and haemorrhage, traumatic brain injury, cardiopulmonary resuscitation (CPR) and post-cardiac management, and peri-operative hyperoxia. Last, the role of SpO_2_ vs. PaO_2_ measurements for the monitoring of oxygenation will be addressed.

## General ICU and emergency department (ED) populations

A retrospective multicentre analysis of patients with an ICU stay > 24 h showed that the "*hyperoxaemia dose*", defined as the time integral of supraphysiological PaO_2_ (> 100 mmHg) was associated with mortality in ICU patients. Interestingly, however, no dose–response relationship could be established [22]. In line with these findings, a retrospective analysis of more than 25,000 mechanically ventilated ICU patients found that the proportion of time spent at 95 ≤  SpO_2_ ≤ 99% was associated with the lowest odds ratio for mortality, while both SpO_2_ ≤ 94 and = 100% coincided with increased mortality [23]. Similarly, an observational study analysing large ICU data bases of a total of 35,287 patients identified 94 ≤  SpO_2_ ≤ 98% as the optimal range with respect to survival [24]. In fact, targeting this interval, i.e. "*conservative*" oxygen therapy (PaO_2_ = 70–100 mmHg or SpO_2_ = 94–98%) vs. "*standard*" treatment (PaO_2_ ≤ 150 mmHg or SpO_2_ ≤ 98%) in a general ICU population of 434 patients with an expected length of stay of ≥ 72 h, had previously been associated with significantly reduced mortality (11.6 vs. 20.2%), de novo occurrence of shock (3.7 vs. 10.6%), liver failure (1.9 vs. 6.4%), and bacteraemia (5.1 vs. 10.1%). However, while the originally planned sample size had been 660 patients, the study was stopped early due to difficulties in enrolment after inclusion of 480 patients [25]. Moreover, only 2/3 of the patients needed mechanical ventilation, and only 30% of the patients presented with shock at inclusion [25].

However, another retrospective single-centre study in general ICU patients mechanically ventilated for at 7 days did not show any association between in-hospital mortality and time-weighted PaO_2_ > 120 mmHg [26]. Furthermore, other authors had even reported nadir mortality in a general ICU population at a mean PaO_2_ over the total ICU length of stay ≈ 120–150 mmHg, while exposure to PaO_2_ > 200 mmHg was indeed associated with increased mortality [27].

An observational cohort study analysed the outcome of patients and exposed to ED hyperoxia. The study included 688 out of a total of 3525 patients already mechanically ventilated in the ED, who were all normoxic (60 ≤ PaO_2_ ≤ 120 mmHg) on day 1 of their ICU stay. While ED normoxia was present in 50.9% of the patients, ED hyperoxia as defined as a PaO_2_ > 120 mmHg occurred in 43.6%. Hospital mortality at day 28 was higher in patients with ED hyperoxia (29.7%) than in those with ED normoxia (19.4%) [28]. Interestingly, survival curves of patients with ED hyperoxia and normoxia, respectively, cleaved at day 4–5 only of hospital stay, i.e. several days after normoxia had resumed.

In conclusion, in general ED and ICU populations, so far, the available clinical data do not suggest that there is any ideal target PaO_2_ except for avoiding prolonged exposure (> 24 h) to either hypoxemia or supraphysiological (PaO_2_ > 100 mmHg).

## Acute respiratory distress syndrome (ARDS)

The current standard of care of patients with ARDS recommends targeting for arterial oxygenation defined as PaO_2_ = 65–75 mmHg and/or arterial haemoglobin O_2_ saturation (SaO_2_) = 90–95%, respectively [29]. In a secondary analysis of 2005 patients of the LUNG SAFE study, Madotto et al*.* reported an incidence of "*hyperoxaemia*" (PaO_2_ > 100 mm Hg) on day 1 or "*sustained hyperoxaemia*" (i.e. on day 1 *and* 2) in 30 and 12% of the patients, respectively. In 66% of these hyperoxaemic patients, "*excess O*_*2*_* use*", i.e. an inspiratory O_2_ concentration F_I_O_2_ ≥ 0.6 *and* PaO_2_ > 100 mmHg, was present. The authors concluded that despite being frequently present, hyperoxaemia was mostly only transient. However, neither hyperoxaemia nor excess O_2_ use had any effect on patient outcome [30].

The LOCO2 trial [31] compared "*conservative O*_*2*_" therapy for 7 days (target PaO_2_ = 55–70 mm Hg and/or SaO_2_ as measured by pulse oximetry [SpO_2_] = 88–92%) to a "*liberal O*_*2*_" therapy arm (target PaO_2_ = 90–105 mm Hg and/or SpO_2_ ≥ 96%). Albeit the patients in the liberal O_2_ arm showed significantly higher PaO_2_ values than those in the conservative O_2_ group without major inter-group overlap, about half of the patients of the conservative O_2_ group presented with values beyond the upper target threshold of 70 mmHg. Nevertheless, during the 7 observation days, the conservative O_2_ group needed less controlled ventilation, lower PEEP levels, and prone positioning. In contrast, heart rate was higher in this group. However, the conservative O_2_ approach did not increase survival at 28 days, and the trial was prematurely stopped because of safety concerns after enrolment of 205 patients: at day 28, 34 of 99 patients (34%) in the conservative and 27 of 102 patients (26.5%) in the liberal O_2_ groups, respectively, had died. At day 90, mortality was even significantly higher in the conservative (44 of 99 [44%] patients) than in the liberal O_2_ arm (31 of 102 [30%] patients) [31].

Finally, in a total of 2,928 patients with acute hypoxemic respiratory failure, the most recent ICU-HOT trial compared a "*lower*" (PaO_2_ ≈ 60 mmHg) *vs.* a "*higher*" (PaO_2_ ≈ 90 mmHg) oxygenation target until 90 days after randomization [32]. More than half of the patients needed mechanical ventilation and/or vasopressor support (58 and 54%, respectively). Interestingly, in both study groups, the median/interquartile range PaO_2_ values were slightly higher than the target levels (71/67–77 vs. 93/87–99 mmHg, respectively). The two intervention groups did not show any difference with respect to mortality and morbidity.

In conclusion, in patients with ARDS, so far, the available clinical data do not suggest that there is any ideal target PaO_2_ except for avoiding prolonged exposure (> 24 h) to either hypoxemia, in particular with respect to long-term neuropsychological sequelae [33, 34], or supraphysiological (PaO_2_ > 100 mmHg).

## Sepsis and septic shock

Hyperoxaemia is associated with systemic vasoconstriction, i.e. might theoretically counteract vasodilation-related arterial hypotension [5], and has antibacterial potential [35]. However, in a retrospective analysis of 141 patients (out of a total enrolment of 503 patients) with ventilator-associated pneumonia (VAP), the number of days with hyperoxaemia (as defined by a PaO_2_ > 120 mmHg) was an independent risk factor for the occurrence of VAP, rather than suggesting any antibiotic property. It should be noted, however, that these patients also showed several other risk factors of VAP, e.g. more use of proton pump inhibitors, more frequent circulatory shock, more transfusion of packed red blood cells, and more frequent sedation [36].

The prospective, randomized, controlled HYPER2S trial [37] compared standard therapy with mechanical ventilation with 100% O_2_ during the first 24 h after diagnosis of septic shock. Despite a significantly lower SOFA score at day 7, the trial was prematurely stopped due to increased mortality in the treatment arm at days 28 and 90 (43 vs. 36 and 48 vs. 42%, respectively). Due to the premature stop of the trial, this increase in mortality did not reach statistical significance (*p* = 0.12 and 0.16, respectively) [37]. The detrimental effect of hyperoxia was further mirrored by a higher incidence of serious adverse events (85 vs. 76%, *p* = 0.02), in particular of ICU-acquired weakness (11 vs. 6%, *p* = 0.06). Of note, a post hoc analysis according to the Sepsis-3 criteria showed that the hyperoxia-related increase in mortality at day 28 was only present in patients with hyperlactataemia > 2 mmol/L (57% vs. 44%, *p* = 0.054), while no effect was present in patients with normal lactataemia (25 vs. 23%, *p* = 0.68) [38]. This finding suggests that the putative hyperoxaemia-related increase in tissue O_2_ availability may have led to excess ROS production and consecutive oxidative damage because of a sepsis-induced impaired cellular O_2_ extraction.

The above-mentioned "*conservative*" oxygenation target in the ICU-ROX study was not beneficial either in patients with sepsis: a post hoc analysis of the 251 patients fulfilling these criteria showed no statistically significant inter-group difference when compared to a "*usual*" oxygenation. Point estimates of treatment effects even consistently favoured the latter [19]. Hence, similar to ARDS, in patients with sepsis/septic shock, there seems to be no ideal target PaO_2_ except for avoiding prolonged exposure (> 24 h) to either hypoxemia or supraphysiological (PaO_2_ > 100 mmHg).

## Trauma and haemorrhage

The blood loss-related reduction of O_2_ transport capacity and the fall in cardiac output during trauma and haemorrhage cause a tissue O_2_ debt, the rapid repayment of which determines outcome [39]. There is experimental evidence that during haemorrhage, the hyperoxia-related rise in PaO_2_ can improve microcirculatory and tissue O_2_ availability [40]. Moreover, lung-protective ventilation with an F_I_O_2_ = 1.0 even attenuated organ dysfunction in resuscitated, long-term large animal experiments [41, 42]. Nevertheless, clinical data remain equivocal. In 471 consecutive mechanically ventilated patients with a median injury score (ISS) of 29, Russell et al*.* reported that there was no association between mortality and maximum PaO_2_ in the first 24 h. This was true both for the overall analysis as well as in the subgroup with head trauma (n = 266) [43].

More recently, Baekgaard et al*.* reported on 5,912 patients of a French trauma registry (median ISS 16), 32% of whom presented with traumatic brain injury (TBI). Univariate analysis showed *higher* mortality (12 vs. 9%, *p* < 0.0001) in patients with hyperoxaemia as defined by PaO_2_ > 150 mmHg upon admission (43% of the population). However, propensity score matching for gender, age, prehospital heart rate and systolic blood pressure, temperature, haemoglobin and arterial lactate concentration, use of mechanical ventilation, presence of traumatic brain, initial Glasgow Coma Scale (GCS) score, ISS, American Society of Anesthesiologists physical health class > I, and presence of haemorrhagic shock yielded just the opposite result, i.e. hyperoxaemia was associated with even *lower* mortality [8]. Most recently, a large retrospective, propensity-matched, data analysis of a total of 864,340 trauma patients (median ISS = 9) investigated the possible association between supplemental O_2_ administration in the ED and in-hospital mortality and development of ARDS. In all three patient categories as predefined according to SpO_2_ < 94%, 94 ≤  SpO_2_ ≤ 97%, and SpO_2_ > 97%, respectively, supplemental O_2_ was associated with a significantly increased odds ratio of both mortality and incidence of ARDS, no matter the presence or absence of TBI [44]. Unfortunately, the authors did not provide information on the presence of circulatory shock nor the need for mechanical ventilation. Finally, a post hoc analysis of the Focused Outcomes Research in Emergency Care in Acute Respiratory Distress Syndrome, Sepsis and Trauma (FORECAST) on 240 patients with ISS ≥ 16 studied the impact of hyperoxaemia during resuscitation (defined as PaO_2_ of ≥ 300 mmHg on hospital arrival and/or 3 h after arrival). The results highlighted the importance of the need for mechanical ventilation and/or the presence/absence of circulatory shock: hyperoxaemia was associated with prolonged ICU sty in patients not intubated in the ED, while no effect was present in mechanically ventilated patients [45]. Of note, unadjusted baseline characteristics not only showed a higher proportion of mechanically ventilated patients, but also significantly lower GCS (6 *vs.* 14) as well as more frequent need craniotomy and transfusion of blood products in the hyperoxaemic group [45].

Consequently, despite being common practice in daily care, in particular in patients with pronounced blood loss, so far, no specific target for PaO_2_ is available.

## Traumatic brain injury

Two opposing statements (*"…The emerging clinical experience demonstrates that hyperoxia is safe and beneficial to the brain…"* [46] and *"…Hyperoxia may be associated with increased mortality in patients with … traumatic brain injury…"* [47]) highlight the discussion on the impact of supraphysiological PaO_2_ in TBI patients during the last decade. This controversy is further mirrored by the results of three clinical studies: In 193 TBI patients (40% with polytrauma) with a GCS of 4 ± 2, Asher et al*.* reported that maximum values of 250 < PaO_2_ < 450 mmHg during the first 72 h were associated with improved outcome [48]. In contrast, Rincon et al*.* reported in 1,212 mechanically ventilated TBI patients (57% with a GCS < 8) that PaO_2_ ≥ 300 mmHg during the first 24 h significantly increased mortality when compared to 60 ≤ PaO_2_ < 300 mmHg (33% vs. 23%) [13]. Finally, Raj et al*.* reporting on 1,116 moderate-to-severe (GCS 3–12) patients with TBI concluded that a PaO_2_ > 100 mmHg within the first 24 h of ICU admission had no predictive value for 6-month mortality [49].

Theoretically, hyperoxaemia may exert beneficial effects during the acute management of traumatic brain injury (TBI) as a result of the above-mentioned vasoconstriction, which would allow for reducing intracranial pressure without compromising tissue oxygenation. This potentially beneficial effect has been demonstrated in severe TBI patients (*n* = 42, mean GCS = 5.7) undergoing a combined hyperbaric oxygen (HBO) and normobaric (NBO) oxygen treatment. Ultimately, this approach not only significantly reduced mortality, but also improved neurological outcome at 6 months post injury as assessed using the Glasgow Outcome Scale-Extended (GOSE) [50].

In the context of the above-discussed hyperoxaemia-related increase of systemic O_2_ transport capacity, hyperoxaemia may be particularly important when TBI coincides with haemorrhage. Nevertheless, the existing data of the two existing prospective clinical studies again are equivocal: Taher et al*.* compared the effects of 6 h of F_I_O_2_ = 0.8 and 0.5 early after injury in 68 mechanically ventilated TBI patients with a GCS of 4 ± 2, demonstrating a tendency towards improved neurological outcome at 6 months [51]. However, the generalizability of the findings is limited, since patients with chronic co-morbidity,  > 65 years of age, and, in particular, arterial hypotension were excluded.

The BRAINOXY [52] trial investigated the effect of F_I_O_2_ = 0.7 vs. 0.4 in 62 TBI patients (both isolated TBI and TBI in the context of polytrauma) during the time of mechanical ventilation (up to 14 days). As expected, the higher F_I_O_2_ was associated with nearly twice as high PaO_2_ levels during the observation period, but there was no inter-group difference for markers of oxidative stress, inflammation, neurological injury and/or pulmonary complications [52].

Large scale, retrospective studies provided comparably equivocal results: O’Briain et al*.* reported in > 24,000 mechanically ventilated TBI patients that hyperoxaemia (in PaO_2_ category increments up to > 500 mm Hg) during the first 24 h in the ICU did not affect mortality irrespective of the GCS at admission [53]. In contrast, a post hoc analysis of 417 of the 1213 patients of the Citicoline Brain Injury Treatment Trial (COBRIT) showed that 150 < PaO_2_ < 250 mmHg (referred to by the authors as "*mild*" hyperoxaemia) within the first 24 h after injury was associated with significantly lower mortality and, in particular, a better GOSE score and overall long-term functional and cognitive outcomes [9]. This possibly beneficial effect was also suggested by a retrospective study in 115 patients with severe TBI, which assessed the possible relation between PaO_2_ and cerebral metabolism as well as pressure-flow autoregulation using cerebral microdialysis and the pressure reactivity index, respectively. The authors concluded that a PaO_2_ "…*above 90 mmHg and higher may improve oxidative cerebral energy metabolism and pressure autoregulation, particularly in cases of limited energy substrate supply in the early phase of TBI*…". However, there was no significant relationship between PaO_2_ and clinical outcome as assessed using the GOSE [54].

In conclusion, it seems to be unequivocal that hyperoxaemia should be avoided after acute brain injury resulting from ischaemia [13] as well as subarachnoid [55] and/or intracerebral bleeding: Because of the vasoconstrictor properties of O_2_ [5] there is a consecutive risk of vasospasm-induced delayed cerebral ischaemia [56, 57]. Given the above-mentioned controversial results reported on TBI, the current knowledge on a possible PaO_2_ target in these patients is best characterized by the statements that—with respect to any possible neuroprotective properties of hyperox(aem)ia—there *"… is probably a narrow effective dose, and benefit may be limited to at-risk tissue…"* [58], and the conclusion that there is the *" … need to identify optimal approaches to improve O*_*2*_* delivery without exacerbating … oxidative stress or injury…"* [59].

## Cardiopulmonary resuscitation (CPR) and post-cardiac management

CPR represents whole-body I/R injury, and clearly, two phases of CPR have to be differentiated, i.e. the period of active cardiopulmonary resuscitation, and the post-resuscitation phase, which starts after return of spontaneous circulation (ROSC). For obvious reasons, studies on the impact of hyperox(aem)ia during the former are scarce given the common practice of ventilation with 100% O_2_ *during* CPR, but the available data suggest that there is a "*dose-dependent*" successful incidence of ROSC with incremental PaO_2_ values [60, 61].

The post-resuscitation phase has been thoroughly investigated during the last decade. Two large-scale retrospective American and Australian studies including 6,326 and 12,108 patients, respectively, found equivocal impact of arterial oxygenation on outcome after cardiac arrest. In the American study [10], hyperoxaemia (PaO_2_ ≥ 300 mmHg during the first of 24 ICU hours after ROSC) was associated with a significant, dose-dependent increase of mortality and worsening of neurological outcome for any 100 mmHg rise in PaO_2_ [62]). In contrast, in the Australian study, it did not "*have a robust or consistently reproducible association with mortality*" [11]. Similar deleterious effects of hyperoxaemia as defined by PaO_2_ > 300 mmHg have been reported by others in retrospective analyses of a total of 1,448 patients [14, 63–65]. Of note, one of these studies concluded that the *"…optimal range of PaO*_*2*_* for favourable neurological outcome…*" could be a PaO_2_ interval of 70 < PaO_2_ < 240 mmHg [64]. In addition, in the other study, "*moderate hyperoxia*" (101 < PaO_2_ < 299 mmHg) according to the authors’ definition was even associated with improved organ function at 24 h [65]. Finally, in 5,258 patients after ROSC, Helmerhorst et al*.* had observed a similar U-shaped relation between mortality and the maximum PaO_2_ during the first 24 h in the ICU [66] as in the above-discussed general ICU population, the nadir mortality coinciding with a PaO_2_ ≈ 150–200 mmHg.

The most recent data confirm these conflicting results: In a prospective observational study on 280 patients Roberts et al*.* [67] concluded that PaO_2_ > 300 mmHg during the initial 6 h after ROSC was independently associated with death and poor neurological function. Moreover, any one-hour longer duration of hyperoxaemia was associated with a 3% increase in risk of poor outcome. In contrast, a post hoc sub-study of the Target Temperature Management (TTM) trial, including 939 patients after out-of-hospital-cardiac arrest, did not find any significant relation between hyperoxaemia (defined as PaO_2_ > 300 mmHg) within 37 h of ROSC and poor neurological outcome after 6 months [68]. Finally, an additional post hoc analysis of the above-mentioned ICU-ROX study including 161 patients with "*suspected hypoxic–ischaemic encephalopathy*" after cardiac arrest, a subgroup pre-specified and defined prior to randomization, did not show a statistically significant reduction in death or unfavourable neurological outcomes at day 180 [69]. This finding was in contrast to the analysis of the complete GOSE, which had suggested benefit of the "conservative oxygen" treatment and yielded significantly lower mortality at day 180 in the unadjusted analysis [18].

Again, as already suggested for ARDS, sepsis and septic shock, there seems to be no ideal target PaO_2_ for ROSC after CPR.

## Peri-operative hyperoxia

Despite the recommendations of the World Health organization from 2016 [70] and subsequently by the US Centers for Disease Control in 2017 [71], the question whether or not there is evidence for the use of peri-operative hyperoxia to reduce surgical site infections remains a matter of debate. In line with previous ones [72, 73], the most recent meta-analysis on 17 RCT including 7,817 patients identified a weak signal (Odds ratio 0.80, confidence interval 0.64–0.99) favouring peri-operative hyperoxia in patients undergoing general anaesthesia with endotracheal intubation [74]. No effect was found in the overall analysis. Other authors cautioned this conclusion [75] or even could not confirm this result when analysis "…*was restricted to objective- or investigator-identified low-bias studies, although those analyses were not as well-powered*" [76]. Moreover, several concerns have to be raised concerning these recommendations. Most trials investigating the putative impact of peri-operative hyperoxia on surgical site infection compared F_I_O_2_ = 80% with F_I_O_2_ = 30%), which would yield a PaO_2_ ≈ 350–400 mmHg *vs.* PaO_2_ ≈ 120–150 mmHg, respectively, in patients without major acute or chronic cardiopulmonary disease. However, at least some observations reported standard daily practice frequently using F_I_O_2_ ≈ 40—60% [77, 78]. Finally, even if present, any beneficial effect of peri-operative hyperoxia on the incidence of surgical site infections has to be weighed by potential harm, e.g. pulmonary and/or cardiovascular complications as well as new or recurrent cancer [79–83]. In fact, the largest available study in this context on almost 74,000 patients, showed a dose-dependent increase in major post-operative respiratory complications and ultimately 30-day mortality of intra-operative F_I_O_2_ increments (median F_I_O_2_ = 0.3, = 0.41, = 0.52, = 0.58, and = 0.79, respectively) [84].

Hence, albeit a recent review article conjected "…*that current evidence is in favour of hyperoxia in noncritically ill intubated adult surgical patients…*" [85], peri-operative hyperoxia (and even the possible consecutively enhanced host-defence resulting from increased ROS formation [86]) remains an open question, because the "*good*", "*bad*", and "*ugly*" [87] of its use are still not fully understood.

## SpO_2_ vs. PaO_2_ measurement for monitoring of oxygenation

As described in detail above, there is no ideal PaO_2_ target for critically ill patients so far, no matter the underlying aetiology. Hence, both *hyperoxaemia* (i.e. supraphysiological PaO_2_) *and*, more importantly, due to its short and long-term consequences, *hypoxemia* (i.e. PaO_2_ < 55–60 mmHg) should be avoided. For daily practice, this raises the question whether SpO_2_ suffices as a surrogate for PaO_2_. Hyperoxaemia and hypoxemia can be defined as PaO_2_ > 100 and < 55–60 mmHg, respectively. However, due to the sigmoid haemoglobin–O_2_ dissociation curve and its dependency on pH, PCO_2_, temperature, and erythrocyte 2,3-diphosphoglycerate concentration, the PaO_2_–SaO_2_ relation may vary. Moreover, frequently the available SpO_2_ devices cannot take into account increased met- or carboxy(CO)-haemoglobin levels or the interference of jaundice, because measurements are based on two wavelengths only. Hence, even normal SpO_2_ readings of 94% cannot exclude hypoxemia with PaO_2_ < 60 mmHg [88]. This is particularly important in the most severe patients, when vasoactive drugs must be used [89, 90]. To avoid the risk of hypoxaemia, and taking in account both the potential discrepancies between SpO_2_ and SaO_2_ as well as the above-discussed data, targeting 95 ≤  SpO_2_ ≤ 98% appears to be reasonably safe when PaO_2_ and/or SaO_2_ measurements are not available.

## Conclusion

Several authors reported that in ICU patients there is a U-shaped relation between PaO_2_ and mortality/morbidity. Interestingly, these mostly retrospective studies found that the lowest mortality was present at PaO_2_ ~ 150 mm Hg. Nevertheless, such supraphysiological PaO_2_ values cannot be recommended in general, since the absence or presence of circulatory shock and/or the aetiology of tissue dysoxia, i.e. whether it is mainly due to impaired (macro- and/or microcirculatory) O_2_ transport and/or disturbed cellular O_2_ utilization may determine whether any supraphysiological PaO_2_ level is really beneficial and/or even causes deleterious side effects.

## Data Availability

Not applicable.

## Abbreviations

O_2_: Oxygen
F_I_O_2_: Inspiratory O_2_ concentration
PaO_2_: Arterial O_2_ tension
ROS: Reactive oxygen species
I/R: Ischaemia/reperfusion
ATP: Adenosine triphosphate
ICU: Intensive care unit
SpO_2_: Pulse oximetry haemoglobin O_2_ saturation
ED: Emergency department
ARDS: Acute respiratory distress syndrome
TBI: Traumatic brain injury
CPR: Cardiopulmonary resuscitation
SaO_2_: Arterial haemoglobin O_2_ saturation
VAP: Ventilator-associated pneumonia
SOFA: Sequential organ failure assessment
ISS: Injury severity score
HBO: Hyperbaric oxygen
NBO: Normobaric
GCS: Glasgow Coma Scale
GOSE: Glasgow Outcome Scale-Extended
ROSC: Return of spontaneous circulation
RCT: Randomized controlled trial
